# Food introduction during the first year of life in a Swedish birth cohort – associations with allergy at 6 years of age

**DOI:** 10.1186/s12937-026-01344-4

**Published:** 2026-06-17

**Authors:** Mia Stråvik, Anna Sandin, Bill Hesselmar, Agnes E. Wold, Malin Barman, Ann-Sofie Sandberg

**Affiliations:** 1 Department of Life Sciences, Food and Nutrition Science, Chalmers University of Technology, Gothenburg, Sweden; 2 Department of Clinical Science, Pediatrics, Sunderby Research Unit, Umeå University, Umeå, Sweden; 3 Department of Paediatrics, Institute of Clinical Sciences, Sahlgrenska Academy, University of Gothenburg, Gothenburg, Sweden; 4 Department of Infectious Diseases, Institute of Biomedicine, Sahlgrenska Academy, University of Gothenburg, Gothenburg, Sweden

**Keywords:** Allergic asthma, Atopic eczema, Children, Food allergy, Food introduction, Meat, Timing, Tolerance

## Abstract

**Background:**

Diet contains components that may exert broad immunoregulatory properties, including the promotion of tolerance. The timing of food introduction may influence the development of allergies. The aim of this study was to relate the timing of food introduction with doctor’s diagnosed allergies (food allergy, atopic eczema, and allergic asthma) at six years of age in a cohort of Swedish infants.

**Methods:**

At six years of age, 430 children from the Swedish NICE birth cohort were assessed for food allergies, atopic eczema, and allergic asthma by a pediatric allergologist. Timing of introduction of 18 predefined foods (potatoes and roots, fruits and/or fruit juice, berries, nuts and/or almonds, peanuts and/or peanut oil, bread and/or biscuits, butter, margarine, vegetable oils, cow’s milk, ice cream, sour milk (“fil”), yoghurt, meat, fish, eggs, porridge, and gruel) was collected monthly via parent-completed web-based questionnaires. Associations were examined using logistic regression adjusted for allergic heredity.

**Results:**

During the first year of life, 27% of the children received peanuts and/or peanut oil and 32% nuts and/or almonds. Earlier meat introduction was associated with 24% lower odds of food allergy per month earlier introduced [adjusted OR (95% CI): 0.76 (0.60–0.99), *p* = 0.028]. A similar tendency was observed for eggs, although not surviving adjustment for allergic heredity. Children with food allergies had been introduced to margarine earlier than non-allergic children [9.0 (5.0–12.0) vs. 12.0 (9.0–12.0) months, *p* = 0.021], although this finding likely reflects reverse causation.

**Conclusions:**

Our results suggest that earlier introduction of meat may be associated with a lower risk of food allergies later in childhood, although this finding must be interpreted with caution as causality cannot be proven. Timing of food introduction did not appear to be associated with atopic eczema or allergic asthma. Furthermore, our results indicate that recommendations for introduction of potentially allergenic foods during the first year of life were not effectively implemented.

**Trial registration:**

ClinicalTrials.gov, NCT05809479, 12 April 2023, Retrospectively registered.

**Supplementary Information:**

The online version contains supplementary material available at 10.1186/s12937-026-01344-4.

## Background

The incidence of allergic diseases increased dramatically during the 20th century in Western countries [1], where allergies today represent the most common chronic diseases in children and young adults. Allergies impose substantial burdens on individuals and society, with some allergies causing life-threatening anaphylaxis if left untreated.

In an attempt to halt the rapid increase of food allergies, the American Academy of Pediatrics, in 2000, recommended delaying the introduction of potentially allergenic foods in high-risk families (dairy products ≥ 1 year, eggs ≥ 2 years, fish, peanuts, nuts ≥ 3 years) [2]. However, studies investigating food allergen elimination failed to show a protective effect on allergy development [3, 4], and the recommendations were therefore withdrawn eight years later [5]. A notable observation contradicting the avoidance paradigm was that peanut allergy was ten times more common in children raised in the United Kingdom, where peanuts were avoided during the first year of life, compared to children of the same ancestry raised in Israel, where peanuts were widely consumed during infancy [6].

Based on these findings, the Learning Early about Peanut Allergy (LEAP) randomized controlled trial (RCT) was performed [7]. It showed that continuous consumption of peanuts from four to eleven months of age in high-risk infants with severe eczema and/or egg allergy reduced peanut allergy prevalence at five years of age by 70–86% (non-sensitized and sensitized at start, respectively) compared with total avoidance until five years [7]. Another large RCT, the Enquiring About Tolerance (EAT) study, examined introduction of six common potentially allergenic foods (cow’s milk, eggs, peanuts, wheat, whitefish, and sesame) from three months of age and showed a trend toward reduced peanut and egg allergy in infants fed considerable amounts of these foods [8]. While evidence for allergen introduction is strongest for peanuts and eggs, additional RCTs and observational studies have since then shown that early introduction also of other allergens is associated with lower food allergy risk [9–12].

Apart from potentially allergenic proteins introduced to induce allergen-specific tolerance, complementary foods also contain bioactive components with broad immunoregulatory properties (e.g., n-3 LCPUFAs). For instance, early fish introduction has been associated with reduced risk of allergic diseases, including atopic eczema and asthma, beyond just fish allergy [13–15].

Based on increasing evidence that early food introduction may prevent allergy development, families worldwide are now recommended to introduce complementary foods between four and six months of age [16]. However, recent findings from the PreventADALL trial in a Nordic population suggest that introducing peanuts, cow’s milk, eggs, and wheat as early as three months of age might prevent the respective food allergies [17].

The aim of the present study was to relate the timing of food introduction with doctor’s diagnosed allergies (food allergy, atopic eczema, and allergic asthma) at six years of age in the Swedish NICE (*Nutritional impact on Immunological maturation during Childhood in relation to the Environment*) birth cohort.

## Methods

### Study design

The NICE birth cohort (ClinicalTrials.gov identifier: NCT05809479) included 655 pregnancies with planned deliveries at Sunderby Hospital, Luleå, in northern Sweden, between February 2015 and March 2018. Expecting parents were invited to participate during a routine ultrasound at gestational week 18. Inclusion required written informed consent, mailed by post, and ability to communicate in written and spoken Swedish. More detailed information regarding the NICE cohort can be found in the study protocol [18]. The study was conducted in accordance with the Helsinki Declaration and approved by the Regional Ethical Review Board in Umeå, Sweden (2013/18−31 M, 2015−71−32). Parents were informed of their right to withdraw from the study at any time without stating a reason, and have their data deleted and biological samples discarded.

### Inclusion and exclusion criteria

**Fig. 1:**
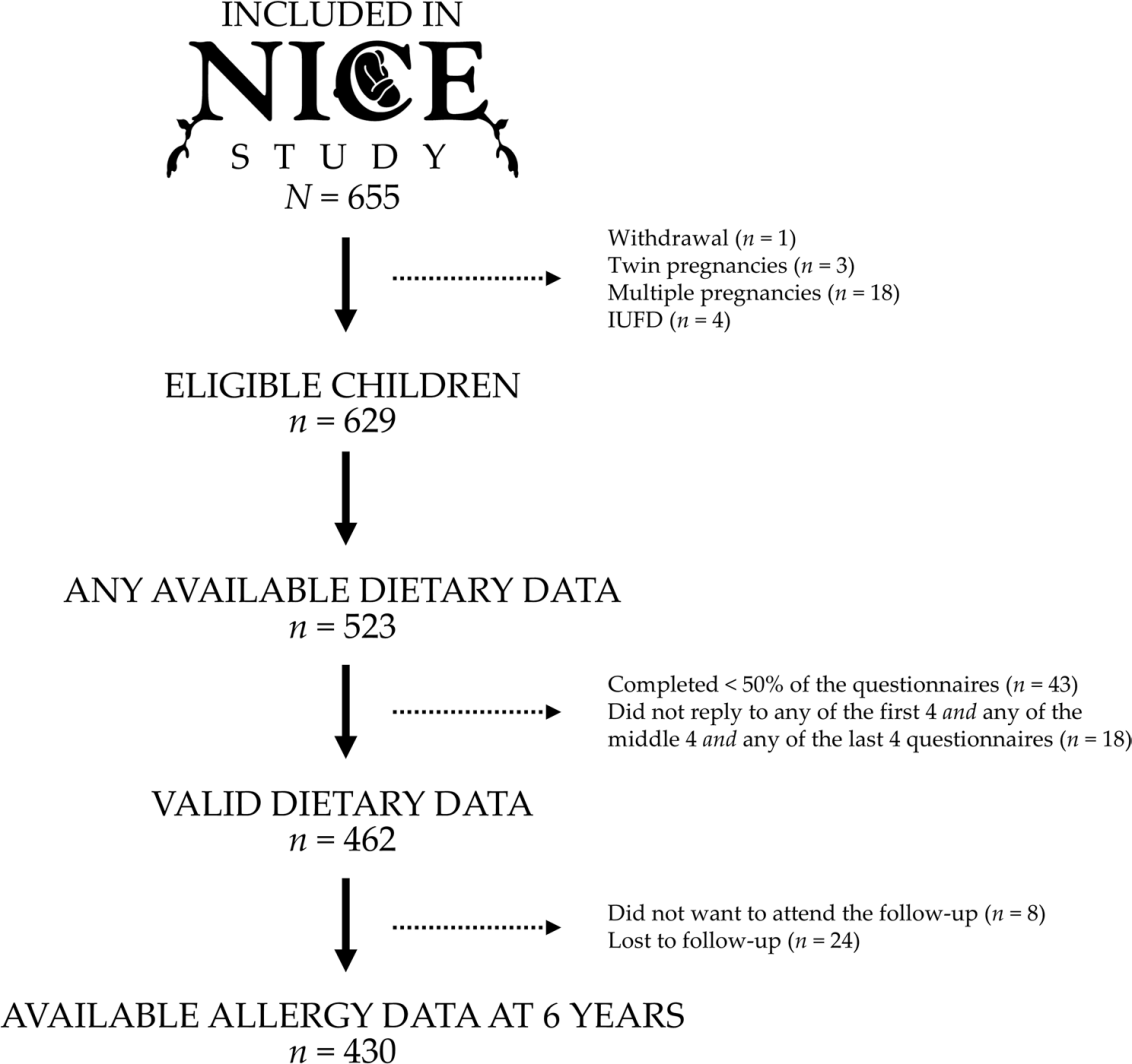
Flowchart of study inclusion and exclusion

Children were eligible for this study on food introduction and allergies if they were liveborn singletons. Further, in families with multiple participating children born during the study period, only firstborn children were included to avoid non-independent observations. Families also had to respond to ≥ 50% of the monthly questionnaires, including at least one response during early (1–4 months), middle (5–8 months), and late (9–12 months) infancy, to capture variation in timing of food introduction. In total, 462 children had valid dietary data and were included in the descriptive analyses of food introduction. Among these, 430 children had allergy data available at six years of age and were included in the analyses relating food introduction to allergic disease (Fig. 1).

### Assessment of breastfeeding, formula feeding, and introduction of complementary foods

During the infant’s first year of life, parents completed monthly web-based questionnaires that included questions about breastfeeding (exclusive or partial) and use of infant formula during the previous month. They were also asked whether any of 18 predefined foods had been introduced during the previous month: 1) potatoes and roots; 2) fruits and/or fruit juice; 3) berries; 4) nuts and/or almonds; 5) peanuts and/or peanut oil; 6) bread and/or biscuits; 7) butter (including blended butter); 8) margarine; 9) oils (olive, rapeseed and/or sunflower); 10) cow’s milk; 11) ice cream; 12) sour milk (“fil”, a Swedish fermented cow’s milk product fermented at a lower temperature than yoghurt); 13) yoghurt; 14) meat; 15) fish; 16) eggs; 17) porridge; and 18) gruel (a liquid food commonly given to Swedish infants, made by boiling cow’s milk with flour – usually oats but sometimes maize or other grains). Further, parents were asked whether the infant had consumed any dietary sources of probiotic bacteria, such as fruit drinks supplemented with probiotic bacteria or probiotic-containing fermented cow's milk products. In addition to the predefined foods, parents were asked to report any other foods their child had consumed more than twice per week. All 12 monthly questionnaires were completed by 47% of the families, 37% completed 10–11, and 16% completed 6–9 questionnaires.

At the 12-month follow-up visit, parents were asked whether their child had received infant formula during the first days of life in the maternity ward.

### Diagnosis of allergic diseases

At six years of age, all families were contacted by phone and asked about any allergic symptoms in their child. If parents reported symptoms or suspected allergies, they were invited to the study clinic for a thorough allergy examination by the study pediatrician, a specialist in pediatric allergology. The diagnostic procedure for allergic diseases in this cohort, previously described in the study protocol [18], is presented in detail below.

#### Food allergy

Food allergy was diagnosed based on a medical history of allergic reaction to a specific food with symptom improvement after elimination of the allergen [19]. The diagnosis was confirmed by at least one food challenge, except in rare cases with a clear history of anaphylaxis, where provocation was deemed unnecessary and potentially unsafe. Sensitization to the specific food allergen supported the diagnosis in some cases but was not mandatory for diagnosis, as it was used primarily to evaluate the feasibility and safety of performing an oral food challenge.

#### Atopic eczema

Atopic eczema was diagnosed according to William’s criteria [20–22]. More specifically, the child had to have itchiness in combination with ≥ 3 of the following: 1) involvement of skin creases (e.g., elbows, knees, ankles, neck, or cheeks); 2) history of asthma, food allergy, or hay fever; 3) history of generally dry skin; and 4) visible eczema in flexural areas, on the cheeks, forehead, or outer limbs.

#### Allergic asthma

Allergic asthma was diagnosed based on a history of wheezing occurring after exposure to an airborne allergen, with clear improvement of symptoms following treatment and/or avoidance of the allergen. Wheezing triggered solely by infection or physical activity was not included.

### Assessment of covariates

At enrollment in the NICE study, expecting parents received an email with a link to a web-based questionnaire about allergies in the family and home environment. The questionnaire included 116–159 questions, depending on the number of follow-up questions, and collected data on e.g., food avoidance, education level, tobacco use, and presence of animals in the home.

Allergic heredity was assessed by the study pediatrician at the 12-month follow-up and included questions on family history of doctor-diagnosed atopic eczema, food allergy, allergic rhinoconjunctivitis, and/or medically treated asthma. At the same visit, the children were also assessed for allergies by the study pediatrician using the same protocol as at the six-year visit.

### Statistical analysis

Data were analyzed using IBM SPSS ver. 28 (IBM, New York, NY, USA) and R ver. 3.6.2 (R Core Team, Vienna, Austria). Ordinal variables for complementary food introduction were created from monthly binary responses, defining age at introduction as the earliest reported month of intake. Free-text responses for unlisted foods were recoded into existing food groups when classification was unambiguous. No introduction during the first year was coded as occurring at 13 months. To note, month of age refers to the number of completed months since birth, while month of life refers to the month currently underway (e.g., first questionnaire = first month of life, zero months of age).

Logistic regression was used to assess associations between timing of complementary food introduction (month, continuous, reversed order) and allergy diagnoses at six years of age. Non-allergic controls were children free of any allergy diagnosis and allergic sensitization. Results were presented crude and adjusted for allergic heredity (parents and/or siblings). Odds ratios (ORs) with 95% profile likelihood confidence intervals (CIs) and Wald test *p*-values were visualized in forest plots. Crude *p*-values are shown in figures, and false discovery rate (FDR)-adjusted *p*-values (Benjamini-Hochberg) are shown in tables.

Univariate comparisons between groups (e.g., early versus late introduction; allergic versus non-allergic) were tested using Fisher’s exact test (binary variables), linear-by-linear association (ordinal variables), and Mann−Whitney U test (continuous variables). *P*-values < 0.05 were considered significant.

#### Sensitivity analysis

Parents may avoid introducing foods commonly associated with allergies (e.g., peanuts, cow’s milk, eggs) to children with prior reactions. Since food allergy was assessed also at one year of age, children with early-onset food allergy could be excluded in sensitivity analyses.

Among the 16 children with food allergy at six years of age, seven had a food allergy diagnosis also at one year, two had possible/uncertain food allergy, six had no food allergy, and one was unknown. Excluding those with food allergy diagnosis at one year of age resulted in the remaining of six children with food allergies, 43 with atopic eczema, and 39 with allergic asthma at six years of age.

## Results

### Breastfeeding and infant formula feeding

**Fig. 2:**
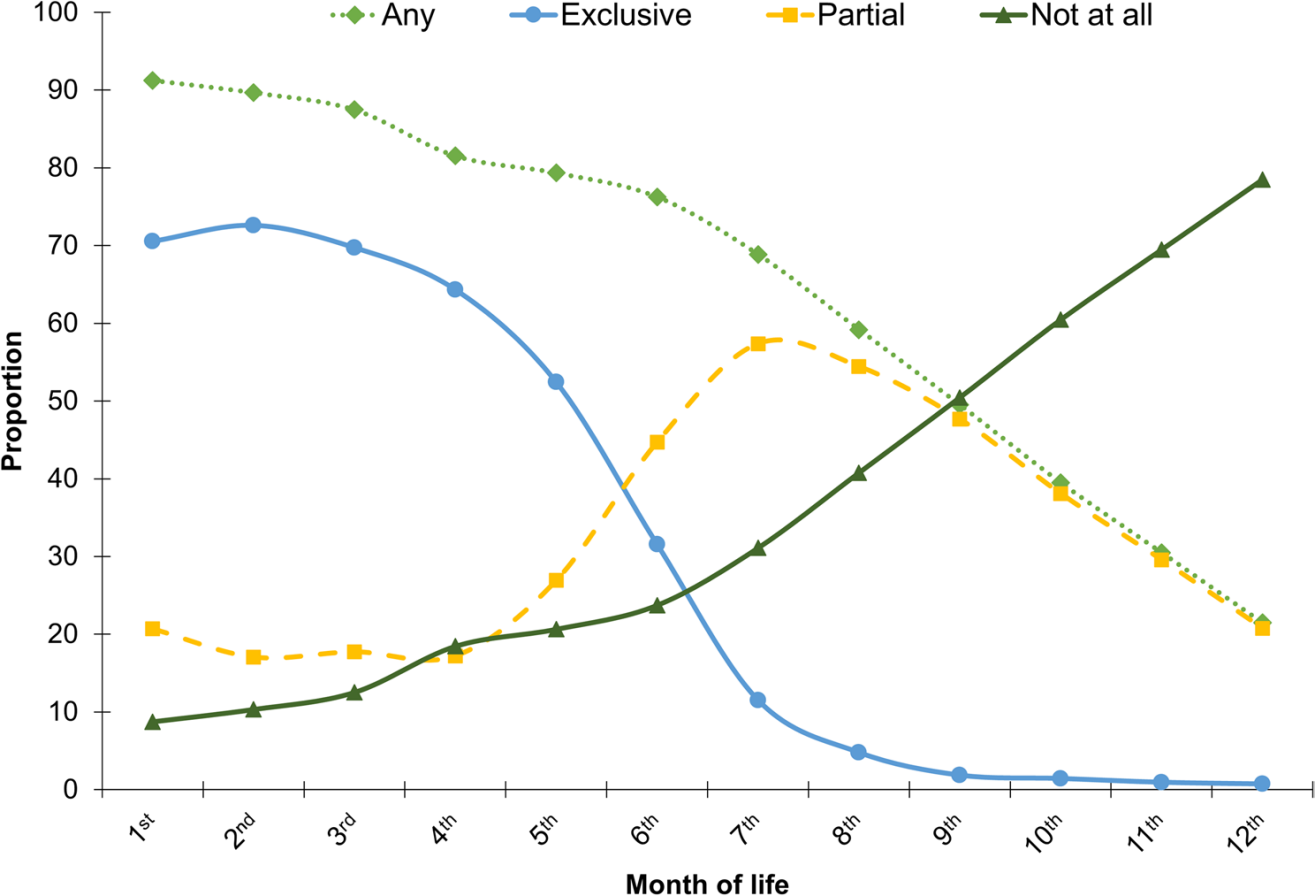
Proportion of children breastfed at each month during the first year of life

The proportion of children breastfed (exclusively or partially) each month is shown in Fig. 2. A majority (91%) were breastfed from birth, with 64% exclusively breastfed at four months. Breastfeeding, regardless of extent, continued to a median of seven months of age.

During the first year, 62% received infant formula (59% cow’s milk-based, 6% cow’s milk-free; Table 1). Infant formula was given to 38% of the children during the first month of life, with 32% being partially formula fed and 6% exclusively formula fed (Supplementary Fig. 1).

**Table 1:** Infant formula use during the first year of life

	All (*n* = 462)	Formula users (*n* = 287)		
	*n* (%)	%	Median (25^th^ – 75^th^ percentiles) ^1^	Description of formula
Cow’s milk-based	273 (59)	95	1 (0–3)	
BabySemp	178 (39)	62	1 (0–4)	0–6 months
NAN Pro 1 or 2	133 (29)	46	2 (0–5)	0–6 and 6–12 months
NAN Sensitive 1	66 (14)	23	1 (0–3)	0–6 months; added lactobacillus
Semper Lemolac	60 (13)	21	2 (0–4)	0–6 months; lower pH
Hypoallergenic	30 (6)	10	2 (1–3)	
Althera	26 (6)	9	2 (1–4)	0–12 months; degraded whey
Nutramigen	5 (1)	2	2 (1–5)	0–12 months; hydrolyzed casein or amino acid based
Neocate	6 (1)	2	4 (3–7)	0–12 months; amino acid based
Other	83 (18)	29	4 (1–6)	

^1^ Month of age when first introduced to the formula. The numbers represent age when consumption occurred, not age when questionnaire was completed (i.e., 0 = introduction in past month before turning one month)

Data on formula use in the maternity ward (most often cow’s milk-based in Norrbotten), were available for 414 (90%) children, of whom 159 (38%) received it there during the first days in life.

### Introduction of complementary foods

**Fig. 3:**
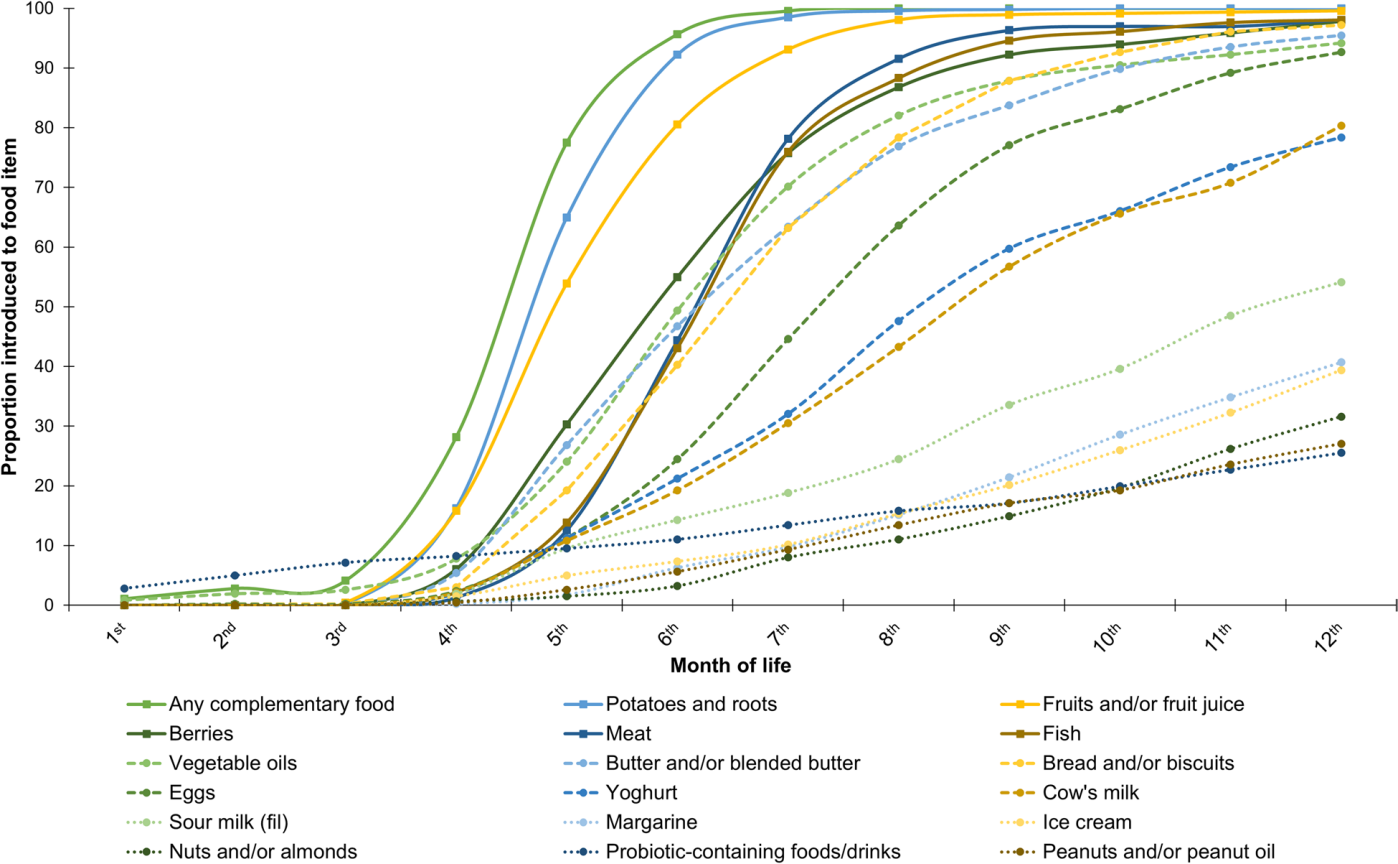
Cumulative proportion of children introduced to complementary foods at each month during the first year of life (*n* = 462)

Complementary foods (apart from infant formula) were introduced at a median age of 4 months (25^th^– 75^th^ percentiles: 3–4 months) with 28% receiving complementary foods before four months, 77% before five months, and 96% before six months of age. All children had been introduced to some type of complementary food before they turned eight months (Fig. 3).

There was a clear order in which complementary foods were introduced, starting with potatoes and root vegetables, and fruits and/or fruit juices (median age: 4 months), followed by berries (5 months), fats (butter and vegetable oils), meat, fish, and bread and/or biscuits (6 months), eggs (7 months), yoghurt and cow’s milk (8 months), and sour milk (11 months).

Potatoes and root vegetables were introduced to all children during the first year, and almost all children received fruits and/or fruit juices (99.6%), berries (98%), meat (98%), fish (98%), bread and/or biscuits (97%), butter and/or blended butter (95%), vegetable oils (94%), and eggs (93%). Four out of five children received cow’s milk (80%) and yoghurt (78%), and every other child received sour milk (54%). Some foods were more rarely introduced during the first year of life: margarine (41%), ice cream (39%), nuts and/or almonds (32%), peanuts and/or peanut oil (27%), and probiotic-containing foods/drinks (26%).

Porridge was introduced earlier than gruel (median age: 4 vs. 6 months) and given to all except two children during the first year. Regular porridge was most common (96%), followed by cow’s milk-free (17% at any time) and gluten-free (15% at any time). Gruel was introduced to 81% of the children during the first year, with regular being the most common choice (77%), followed by gluten-free (13%), cow’s milk-free (9%), lactose-free (2%), and soy-based (1%).

#### Factors associated with introduction of complementary foods

Children were divided into two groups based on complementary food introduction before or at/after four months of age (Table 2). Children with earlier introduction were born at slightly later gestational age (three days), were firstborn (i.e., without older siblings), and more often had a mother who smoked before pregnancy. Maternal smoking remained significant when introduction was handled as a continuous variable (Mann-Whitney U test, *p* = 0.016). Highly educated mothers showed a weak non-significant tendency toward later food introduction, while paternal education was unrelated to timing of food introduction. Allergic heredity did not differ between the groups.

**Table 2:** Family characteristics by timing of food introduction before four months (before median) or from four months of age or later (median and above)

		Age at first food introduction		
	All *n* = 462	< 4 months *n* = 130	≥ 4 months *n* = 332	
Characteristics	*n* (%) or median (25^th^–75^th^ percentiles)			*p*
Birth weight (grams)	3560 (3233–3940)	3613 (3324–3956)	3520 (3210–3935)	0.100
Missing	1	-	1	
Gestational age at birth (days)	281 (275–287)	283 (276–289)	280 (274–286)	0.003
Missing	1	-	1	
Sex (boy)	215 (47)	66 (51)	149 (45)	0.256
Season of birth				
October to March	223 (48)	56 (43)	167 (50)	0.178
April to September	238 (52)	74 (57)	164 (50)	
Missing	1	-	1	
Siblings (in home, full-time)				
Yes	225 (49)	51 (39)	174 (52)	0.013
Allergy diagnosis at 1 year				
Food allergy	35 (9)	12 (12)	23 (8)	0.312
Atopic eczema	30 (8)	15 (15)	15 (6)	0.009
Asthma	27 (7)	8 (9)	19 (7)	0.649
Non-allergic	334 (91)	85 (73)	249 (82)	0.042
Missing	28	10	18	
Allergy in family members				
Mother	178 (41)	53 (44)	125 (40)	0.445
Father	186 (43)	51 (43)	135 (43)	1.000
Sibling	73 (16)	18 (14)	55 (17)	1.000
Any	304 (70)	89 (74)	215 (68)	0.292
Missing	28	10	18	
Residential address				
Town (central part)	196 (46)	52 (44)	144 (46)	0.706
Town (suburb)	106 (25)	30 (25)	76 (24)	
Countryside	127 (30)	36 (31)	91 (29)	
Missing	33	12	21	
Maternal education				
Elementary school, 9 years	7 (2)	4 (3)	3 (1)	0.129
High school, 12 years	127 (28)	39 (30)	88 (27)	
University or other, > 12 years	327 (71)	87 (67)	240 (73)	
Missing	1	-	1	
Paternal education				
Elementary school, 9 years	3 (1)	1 (1)	2 (1)	0.893
High school, 12 years	127 (36)	34 (37)	93 (36)	
University or other, > 12 years	219 (63)	58 (62)	161 (63)	
Missing	113	37	76	
Maternal age (years)	30 (27–34)	30 (27–34)	30 (27–34)	0.685
Maternal smoking pre-pregnancy				
Yes	26 (6)	12 (9)	14 (4)	0.043
Missing	3	1	2	
Mother’s nationality (Swedish)	437 (95)	122 (95)	315 (95)	0.813
Missing	2	1	1	
Early-pregnancy BMI (kg/m^2^)	24.3 (22.0−27.9)	25.0 (22.1–28.2)	24.1 (21.9–27.7)	0.186
Missing	10	3	7	

Participants were divided into two groups based on complementary food introduction (not including infant formula). Differences between groups were tested using Fisher’s exact test (binary variables), linear-by-linear association (ordinal variables), and Mann–Whitney U test (continuous variables)

### Allergy diagnosis at six years of age

**Fig. 4:**
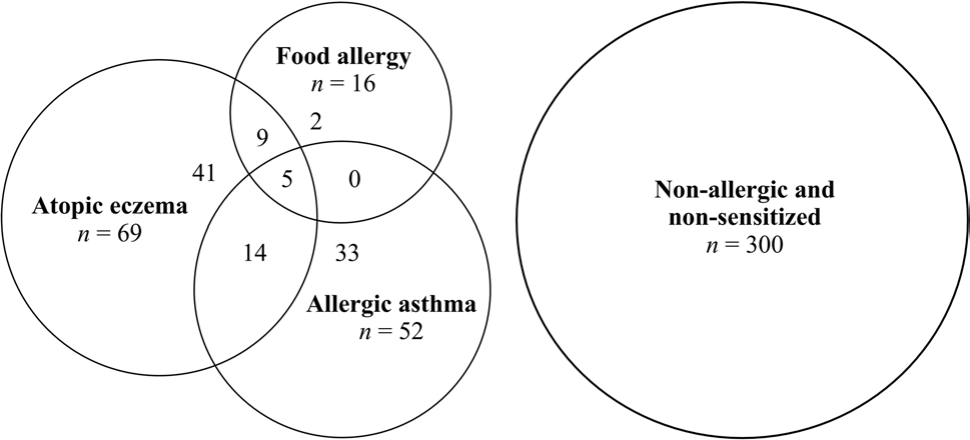
Distribution of children by allergy diagnosis at six years of age

Among the 430 children with both valid dietary data and available allergy data, the prevalence of allergic diseases is shown in Fig. 4. Food allergy, atopic eczema, and allergic asthma were assessed at the six-year follow-up visit by a paediatric allergology specialist.

Food allergy diagnosis was based on elimination-provocation testing, with 3.7% (*n *= 16) diagnosed, of whom seven had multiple food allergies (*n* = 4 reacting to 2 foods, *n* = 2 reacting to 3 foods, *n* = 1 reacting to 4 foods). The offending foods were tree nuts (1.6%, *n* = 7), cow’s milk (1.2%, *n* = 5), egg (0.9%, *n* = 4), peanut (0.9%, *n* = 4), soy (0.7%, *n* = 3), and fish, celery, cinnamon, and kiwi (each 0.2%, *n* = 1).

The prevalence was 16% (*n* = 69) for atopic eczema and 12% (*n* = 52) for allergic asthma. There was a substantial overlap between food allergies and atopic eczema, with 88% (*n* = 14) of those with food allergies also having atopic eczema (Fig. 4).

#### Allergy in relation to breastfeeding

Weaning patterns during the first year of life were compared between infants diagnosed with allergy at six years and those without any allergies (Table 3). No differences were seen in breastfeeding duration between children with and without allergies at six years of age.

**Table 3:** Breastfeeding duration (months) in relation to allergy diagnosis at six years of age

	All children ^1^ Median duration (25^th^−75^th^ percentiles)							
Breastfeeding extent	All children *n* = 462	Non-allergic *n* = 300	Food allergy *n* = 16	*p*	Atopic eczema *n* = 69	*p*	Allergic asthma *n* = 52	*p*
Any	7 (5–10)	7 (5–10)	6.5 (2.25-10)	0.559	8 (5–10)	0.441	7 (4–9)	0.308
Partial	7 (5–10)	7 (5–10)	6.5 (1.5–10)	0.633	8 (5–10)	0.364	7 (3–9)	0.375
Exclusive	4 (2–5)	4 (2–5)	2 (1–4)	0.168	4 (2–5)	0.530	4 (1.25-5)	0.974

	Breastfed children ^2^ Median duration (25^th^ -75^th^ percentiles)							
Breastfeeding extent	All children *n* = 447	Non-allergic *n* = 290	Food allergy *n* = 15	*p*	Atopic eczema *n* = 68	*p*	Allergic asthma *n* = 49	*p*
Any	8 (5–10)	8 (5–10)	7 (3–10)	0.680	8 (5–10)	0.586	7 (5-9.5)	0.431
Partial	8 (5–10)	8 (5–10)	9 (6–10)	0.702	8 (6–10)	0.293	7 (5–10)	0.508
Exclusive	4 (3–5)	4 (3–5)	3 (2–4)	0.140	4 (2–5)	0.806	4 (3–5)	0.486

The numbers represent age when consumption occurred, not age when questionnaire was completed (i.e., 0 = introduction in past month before turning one month). Differences between the non-allergic group (i.e., non-allergic and non-sensitized) and allergy groups were tested using the Mann− Whitney U test

^1^ Includes children who were never breastfed, which may lower the median

^2^ Excludes children who were never breastfed (i.e., duration for breastfed only)

#### Allergy in relation to formula intake

Cow’s milk-free formula was more common among children with allergy diagnoses than non-allergic children, regardless of allergy (i.e., not limited to children with cow’s milk protein allergy): food allergy (25% vs. 4%, *p* = 0.004), atopic eczema (19% vs. 4%, *p* < 0.001), and allergic asthma (19% vs. 4%, *p* < 0.001) (Table 4).

**Table 4:** Infant formula use in the first year and allergy diagnosis at six years of age

	Use of specific infant formula during first year of life						
	Non-allergic (*n* = 300)	Food allergy (*n* = 16)		Atopic eczema (*n* = 69)		Allergic asthma (*n* = 52)	
	*n* (%)	*n* (%)	*p * ^1^	*n* (%)	*p * ^1^	*n* (%)	*p * ^1^
Cow’s milk-based	182 (61)	10 (63)	1.000	39 (57)	0.586	35 (67)	0.440
BabySemp	125 (42)	7 (44)	1.000	20 (29)	0.056	21 (40)	0.880
NAN Pro 1 or 2	93 (31)	4 (25)	0.784	19 (28)	0.664	14 (27)	0.626
NAN Sensitive 1	38 (13)	4 (25)	0.245	11 (16)	0.438	15 (29)	0.005
Semper Lemolac	38 (13)	5 (31)	0.051	11 (16)	0.438	8 (15)	0.655
Hypoallergenic	11 (4)	4 (25)	0.004	13 (19)	< 0.001	10 (19)	< 0.001
Althera	9 (3)	4 (25)	0.002	12 (17)	< 0.001	9 (17)	< 0.001
Nutramigen	2 (0.7)	0 (0)	1.000	1 (1)	0.464	1 (2)	0.382
Neocate	1 (0.3)	1 (6)	0.099	4 (6)	0.005	3 (6)	0.011
Other	58 (19)	3 (19)	1.000	15 (22)	0.619	6 (12)	0.242

^1^ Differences between the non-allergic group (i.e., non-allergic and non-sensitized) and the allergy groups were tested using the Fisher’s exact test

Infant formula use during the first days of life at the maternity ward did not differ between children with and without allergies at six years: 39% (*n* = 106) of non-allergic children versus 33% (*n* = 5) with food allergy (*p* = 0.789), 41% (*n* = 26) with atopic eczema (*p* = 0.776), and 34% (*n* = 17) with allergic asthma (*p* = 0.530).

#### Allergy in relation to timing of food introduction

##### Descriptive analysis of food introduction in relation to allergy

Timing of food introduction was compared between children diagnosed with allergic disease(s) at six years of age and non-allergic, non-sensitized children. Median age at introduction and the 25^th^ -75^th^ percentiles are shown in Supplementary Table 1.

At four months of life (i.e., three months of age), 50% of children later diagnosed with food allergy had received complementary foods, compared to 26% of non-allergic children. By six months of life, all 16 children with food allergies at six years had been introduced to complementary foods. No differences were seen between the non-allergic children and those with atopic eczema or allergic asthma.

**Fig. 5:**
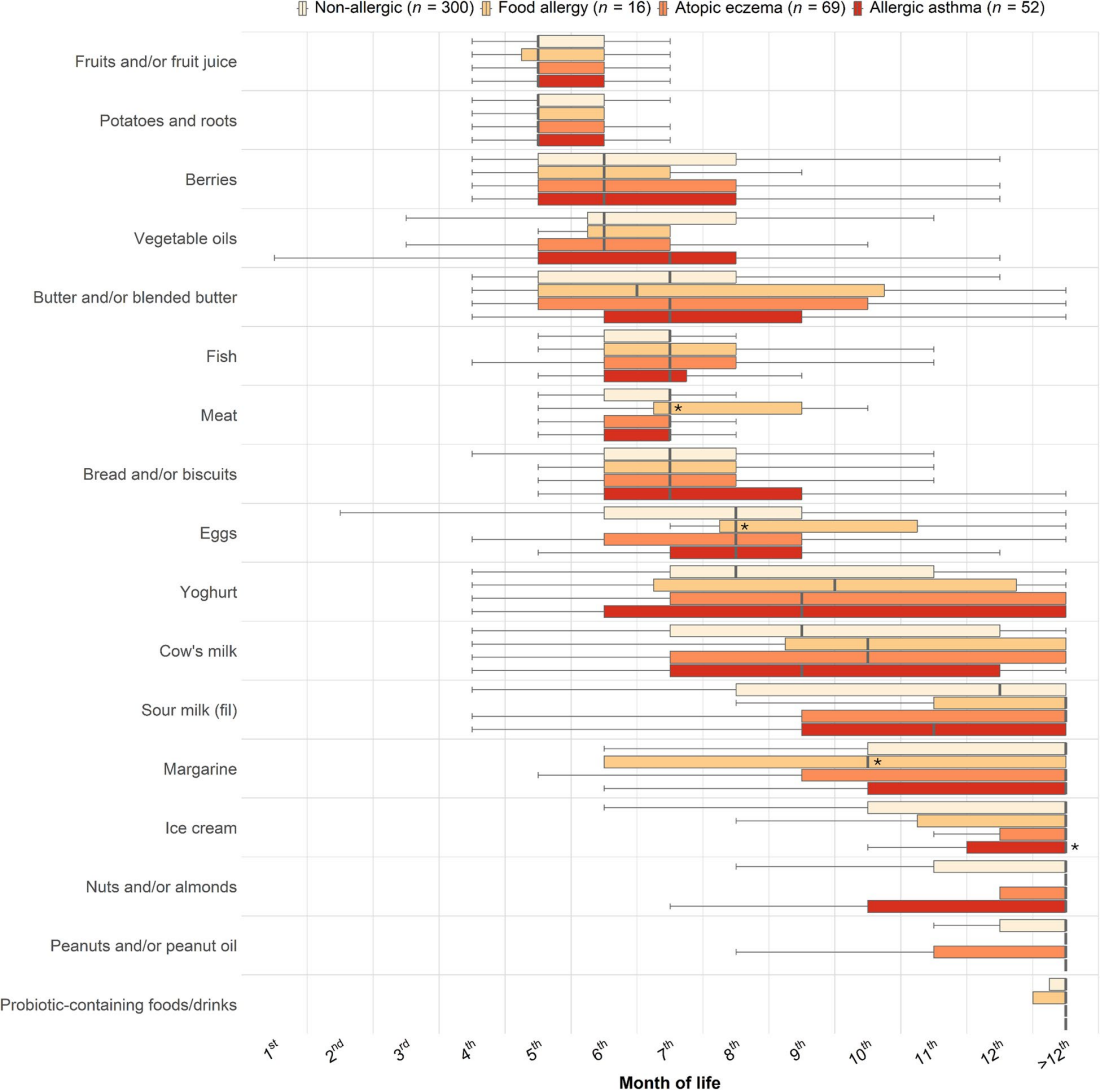
Age at introduction of complementary foods by allergy diagnosis at six years of age. The >12th category indicates that the food was not reported as introduced in any of the 12 monthly questionnaires. The bold vertical line represents the median age at food introduction, and whiskers represent the lowest and highest values within 1.5 × IQR. Differences between each allergy group and non-allergic children were tested using the Mann–Whitney U test, and statistical significance is denoted as: * = *p* < 0.05, ** = *p* < 0.01, *** = *p* < 0.001

Children with food allergies were introduced to meat [median (25^th^ − 75^th^ percentiles): 6.0 (5.25-8.0) vs. 6.0 (5.0–6.0) months, *p* = 0.041] and eggs [7.0 (6.25–11.25) vs. 7.0 (5.0–8.0) months, *p* = 0.049] later than non-allergic children. Conversely, margarine was introduced earlier among children with food allergies [9.0 (5.0–12.0) vs. 12.0 (9.0–12.0) months, *p* = 0.021] (Fig. 5 and Supplementary Table 1).

Food introduction might be postponed due to early allergic reactions (i.e., reverse causation). Therefore, children with food allergy diagnosis at one year were excluded in sensitivity analyses (Supplementary Table 2). Among children with food allergy at six years but without early onset (*n* = 6), meat was still introduced later than in non-allergic children [7.5 (6.0–9.75) vs. 6.0 (5.0–6.0) months, *p* = 0.005]. The median age for egg and margarine introduction were left unchanged but were no longer statistically significant [7.0 (6.75–9.75) vs. 7.0 (5.0–8.0) months, *p* = 0.276; and 9.0 (6.25–12.0) vs. 12.0 (9.0–12.0) months, *p* = 0.098, respectively].

##### Logistic regression analysis of allergy and timing of food introduction

**Fig. 6:**
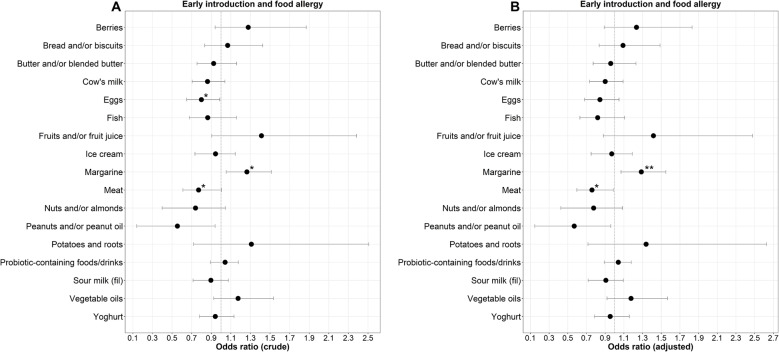
Odds ratios for food allergy at six years of age by timing of complementary food introduction. Logistic regression was conducted with month of introduction (continuous, reversed order) as the explanatory variable and food allergy as the outcome. Dots represent odds ratios (ORs) with 95% profile-likelihood confidence interval error bars (OR < 1 indicates lower odds with earlier introduction). Statistical significance from Wald tests is denoted as: * = *p* < 0.05, ** = *p* < 0.01, and *** = *p* < 0.001. **A**: Unadjusted (*n* = 16 with food allergy, *n* = 300 without any allergies). **B**: Adjusted for allergic heredity (*n* = 15 with food allergy, *n* = 283 without any allergies)

**Fig. 7:**
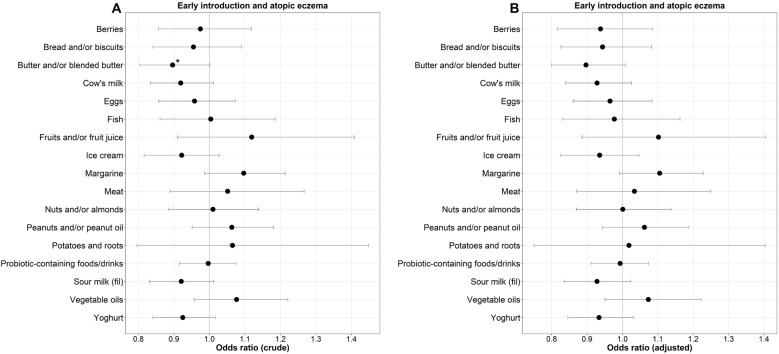
Odds ratios for atopic eczema at six years of age by timing of complementary food introduction. Logistic regression was conducted with month of introduction (continuous, reversed order) as the explanatory variable and atopic eczema as the outcome. Dots represent odds ratios (ORs) with 95% profile-likelihood confidence interval error bars (OR < 1 indicates lower odds with earlier introduction). Statistical significance from Wald tests is denoted as: * = *p* < 0.05, ** = *p* < 0.01, and *** = *p* < 0.001. **A**: Unadjusted (*n* = 69 with atopic eczema, *n* = 300 without any allergies). **B**: Adjusted for allergic heredity (*n* = 66 with atopic eczema, *n* = 283 without any allergies)

**Fig. 8:**
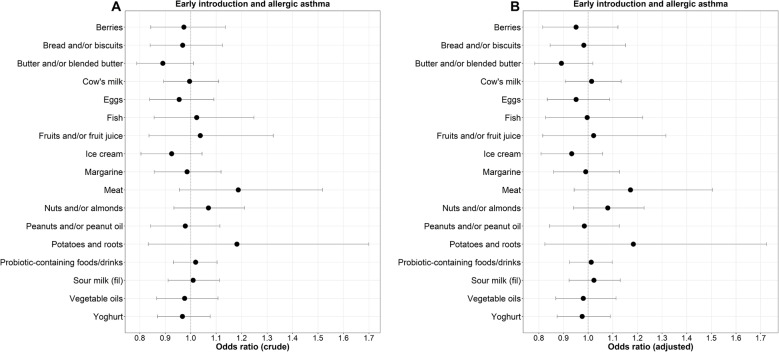
Odds ratios for allergic asthma at six years of age by timing of complementary food introduction. Logistic regression was conducted with month of introduction (continuous, reversed order) as the explanatory variable and allergic asthma as the outcome. Dots represent odds ratios (ORs) with 95% profile-likelihood confidence interval error bars (OR < 1 indicates lower odds with earlier introduction). Statistical significance from Wald tests is denoted as: * = *p* < 0.05, ** = *p* < 0.01, and *** = *p* < 0.001. **A**: Unadjusted (*n* = 52 with allergic asthma, *n* = 300 without any allergies). **B**: Adjusted for allergic heredity (*n* = 51 with allergic asthma, *n* = 283 without any allergies)

To further investigate whether the timing of food introduction is associated with allergic disease, logistic regression analyses were conducted. Food allergy, atopic eczema, and allergic asthma at six years of age were included as binary outcomes in separate models, with timing of food introduction (months of age, reversed order) included as continuous explanatory variables. Results are presented as crude and adjusted for allergic heredity in Figs. 6, 7 and 8. Due to limited sample size, additional confounders were not added to avoid overfitting. Sensitivity analyses excluded children with food allergy diagnosis at one year of age to take reverse causation into account.

Earlier meat introduction was associated with lower odds of food allergy at six years [crude: OR (95% CI): 0.77 (0.61–1.00), *p* = 0.036; adjusted: OR (95%): 0.76 (0.60–0.99), *p* = 0.028] (Fig. 6 and Supplementary Table 3). Excluding children with food allergy diagnosis at one year of age strengthened the association [crude: OR (95% CI): 0.62 (0.45–0.87), *p* = 0.003; adjusted: OR (95%): 0.64 (0.46–0.89), *p* = 0.005].

Introducing margarine earlier was associated with higher odds of food allergy at six years [crude: OR (95% CI): 1.26 (1.05–1.51), *p* = 0.010; adjusted: OR (95%): 1.29 (1.07–1.55), *p* = 0.007]. However, excluding children with food allergy diagnosis at one year left the effect size unchanged but removed significance [crude: OR (95% CI): 1.28 (0.95–1.70), *p* = 0.082; adjusted: OR (95%): 1.27 (0.95–1.68), *p* = 0.086].

Earlier egg introduction was associated with lower odds of food allergy at six years [crude: OR (95% CI): 0.80 (0.65–0.99), *p* = 0.035]. Adjustment for allergic heredity slightly changed the effect size and removed significance [adjusted: OR (95% CI): 0.84 (0.68–1.05), *p* = 0.120], with similar results after exclusion of children with food allergy at one year [crude: OR (95% CI): 0.84 (0.59–1.19), *p* = 0.299; adjusted: OR (95%): 0.85 (0.60–1.19), *p* = 0.320]. To note, none of the associations with food allergy remained statistically significant after adjustment for multiple testing (Supplementary Table 3).

Earlier butter introduction was associated with lower odds of atopic eczema at six years [crude: OR (95% CI): 0.90 (0.80–1.00), *p* = 0.049] (Fig. 7 and Supplementary Table 4). Adjustment for allergic heredity did not change the effect size but removed statistical significance [adjusted: OR (95% CI): 0.90 (0.80–1.01), *p* = 0.066]. The association did not survive adjustment for multiple testing (Supplementary Table 4).

No association was found between timing of food introduction and odds of allergic asthma at six years of age (Fig. 8 and Supplementary Table 5).

## Discussion

This study investigated associations between the timing of complementary food introduction during the first year of life and diagnoses of food allergy, atopic eczema, and allergic asthma at six years of age. Associations were seen between timing of food introduction and food allergy, but not for atopic eczema or allergic asthma.

The strongest association with food allergy was observed for meat, where earlier introduction was linked to lower odds of diagnosis at six years of age. This association remained unchanged after adjustment for allergic heredity. To exclude potential reverse causation (i.e., delayed food introduction due to early allergic symptoms), children diagnosed with food allergy at one year of age were excluded from secondary analyses. The exclusion neither reduced effect size, nor changed the statistical significance. Meat is a rich source of iron, zinc and vitamin A – micronutrients with immunomodulatory functions that, theoretically, may promote oral tolerance [23, 24]. Whether early introduction of meat causally reduces food allergy risk remains to be elucidated, preferably in RCTs. To the best of our knowledge, meat has not been tested in such settings, likely because previous studies hypothesized that introduction of specific allergenic proteins would promote tolerance to those proteins [6–12] - not that foods (e.g., meat) could exert broader tolerance-promoting effects by stimulating immune system maturation, regardless of the allergenicity of the proteins involved.

Another finding, although weaker and without significance after adjustment for allergic heredity, was that earlier egg introduction was associated with lower odds of food allergy diagnosis at six years of age. This finding is, however, supported by previous RCTs [8, 9], potentially suggesting that the lack of statistical significance after adjustment may be due to power issues. For instance, the EAT study showed per-protocol reductions in egg allergy prevalence at three years among children fed eggs (and five other foods) from three months, with higher doses being more protective than lower doses [8]. Another RCT, the PETIT study, showed significant reductions in egg allergy prevalence at one year following a regular egg consumption from six months combined with aggressive eczema treatment [9].

In another cohort, we reported that higher cord serum levels of long-chain polyunsaturated fatty acids might predict later allergy development [25]. Polyunsaturated fatty acids dampen Th1 reactions and T cell proliferation and promote dendritic cells to present antigens in a manner that favors Th2 immune responses [26]. As margarine (with equivalent total fat content as butter) contains 1.7 times more monounsaturated and 4.4 times more polyunsaturated fatty acids than butter [27], it is plausible that margarine given to infants may hamper their immune development and capacity to develop oral tolerance. However, excluding children with food allergies at one year of age removed statistical significance. Thus, it is possible that reverse causation explains this association. More specifically, families might use margarine as a cow’s milk-free butter substitute in children with already developed allergies, since cow’s milk-free formula was given to children with allergies regardless of type, not only cow’s milk protein allergy.

Previous studies have shown associations between maternal margarine intake during pregnancy and lactation [28], and margarine intake in childhood [29], and children’s allergy risk. In this study, we now report a significant association between earlier margarine introduction and food allergy diagnosis at six years. More specifically, the odds of food allergy diagnosis increased by 26–29% per month earlier introduced. While literature on timing remains sparse, one study found that margarine introduction in the first year of life (yes/no) was linked to lower fecal propionate (a short-chain fatty acid) at one year of age, which in turn predicted food sensitization by the age of six years [30].

We found no association between timing of food introduction and allergic asthma at six years of age. Earlier butter introduction was associated with lower odds of atopic eczema, but this did not remain significant after adjustment for allergic heredity. Exclusive or partial breastfeeding duration was not associated with any allergic disease.

Development of oral tolerance requires exposure to potentially allergenic foods. RCTs have clearly shown that early peanut introduction reduces peanut allergy risk [7, 8]. Despite this, Swedish guidelines state that introducing peanuts sometime in the first year can be good, without specifically encouraging earlier introduction [31]. In our study, only one in four children received peanuts and/or peanut oil during the first year, suggesting guidelines did not effectively reach or motivate families to introduce peanuts early on.

A major strength of this study is that allergies were diagnosed by a pediatric allergologist rather than parental report. The same allergologist also assessed the children at one year of age, which enabled investigation of reverse causation in our results. Also, food introduction data were collected monthly, minimizing the risk of recall bias.

However, several limitations must be acknowledged. The monthly questionnaires targeted foods typical for this specific population, potentially limiting generalizability to populations with different feeding practices. Although free-text reporting made it possible to capture unlisted foods (e.g., vegetables, rice, and pasta), distinguishing actual differences in food introduction from parental reporting behavior remains challenging. Further, we did not measure portion sizes, which are crucial since low doses and frequency may be inefficient in promoting oral tolerance [8, 32, 33]. Finally, the moderate sample size limited statistical power, particularly after excluding children with early allergy diagnoses to address reverse causation (i.e., early allergic symptoms making parents reluctant to introduce new foods).

## Conclusions

Our results suggest that earlier introduction of meat may be associated with a lower risk of food allergies later in childhood, although this finding must be interpreted with caution as causality cannot be proven. Timing of food introduction did not appear to be associated with atopic eczema or allergic asthma. Furthermore, our results indicate that recommendations for introduction of potentially allergenic foods during the first year of life were not effectively implemented.

## Supplementary Information

Supplementary Material 1.

## Data Availability

The R-code for conducting the statistical analyses can be obtained from: https://gitlab.com/miastravik/. Data described in the manuscript will not be made available because they relate to information that could compromise research participant privacy or consent. Explicit consent to deposit raw data was not obtained from the participants. Therefore, the data can only be made public if a new consent is filled in by the participants together with a new ethical permit being obtained. Requests to access the datasets should be directed to Ann-Sofie Sandberg, ann-sofie.sandberg@chalmers.se.

## References

[1] Von M. The rising trends in asthma and allergic disease. Clin Experimental Allergy. 1998;28(s5):45–9. 10.1046/j.1365-2222.1998.028s5045.x 9988447

[2] American Academy of Pediatrics. Committee on Nutrition. Hypoallergenic infant formulas. Pediatrics. 2000;106(2 Pt 1):346–9. 10920165

[3] Zutavern A, Brockow I, Schaaf B, von Berg A, Diez U, Borte M, et al. Timing of solid food introduction in relation to eczema, asthma, allergic rhinitis, and food and inhalant sensitization at the age of 6 years: results from the prospective birth cohort study LISA. Pediatrics. 2008;121(1):e44–52. 18166543 10.1542/peds.2006-3553

[4] Snijders BE, Thijs C, van Ree R, van den Brandt PA. Age at first introduction of cow milk products and other food products in relation to infant atopic manifestations in the first 2 years of life: the KOALA Birth Cohort Study. Pediatrics. 2008;122(1):e115–22. 18595956 10.1542/peds.2007-1651

[5] Thygarajan A, Burks AW. American Academy of Pediatrics recommendations on the effects of early nutritional interventions on the development of atopic disease. Curr Opin Pediatr. 2008;20(6):698–702. 19005338 10.1097/MOP.0b013e3283154f88 PMC2659557

[6] Du Toit G, Katz Y, Sasieni P, Mesher D, Maleki SJ, Fisher HR, et al. Early consumption of peanuts in infancy is associated with a low prevalence of peanut allergy. J Allergy Clin Immunol. 2008;122(5):984–91. 19000582 10.1016/j.jaci.2008.08.039

[7] Du Toit G, Roberts G, Sayre PH, Bahnson HT, Radulovic S, Santos AF, et al. Randomized trial of peanut consumption in infants at risk for peanut allergy. N Engl J Med. 2015;372(9):803–13. 25705822 10.1056/NEJMoa1414850 PMC4416404

[8] Perkin MR, Logan K, Tseng A, Raji B, Ayis S, Peacock J, et al. Randomized trial of introduction of allergenic foods in breast-fed infants. N Engl J Med. 2016;374(18):1733–43. 26943128 10.1056/NEJMoa1514210

[9] Natsume O, Kabashima S, Nakazato J, Yamamoto-Hanada K, Narita M, Kondo M, et al. Two-step egg introduction for prevention of egg allergy in high-risk infants with eczema (PETIT): a randomised, double-blind, placebo-controlled trial. Lancet. 2017;389(10066):276–86. 27939035 10.1016/S0140-6736(16)31418-0

[10] Sakihara T, Otsuji K, Arakaki Y, Hamada K, Sugiura S, Ito K. Randomized trial of early infant formula introduction to prevent cow’s milk allergy. J Allergy Clin Immunol. 2021;147(1):224–e328. 32890574 10.1016/j.jaci.2020.08.021

[11] Peters RL, Koplin JJ, Dharmage SC, Tang MLK, McWilliam VL, Gurrin LC, et al. Early exposure to cow’s milk protein is associated with a reduced risk of cow’s milk allergic outcomes. J Allergy Clin Immunology: Pract. 2019;7(2):462–e701. 30267891 10.1016/j.jaip.2018.08.038

[12] Scarpone R, Kimkool P, Ierodiakonou D, Leonardi-Bee J, Garcia-Larsen V, Perkin MR, et al. Timing of allergenic food introduction and risk of immunoglobulin E–mediated food allergy: a systematic review and meta-analysis. JAMA Pediatr. 2023;177(5):489–97. 36972063 10.1001/jamapediatrics.2023.0142 PMC10043805

[13] Hesselmar B, Saalman R, Rudin A, Adlerberth I, Wold A. Early fish introduction is associated with less eczema, but not sensitization, in infants. Acta Paediatr. 2010;99(12):1861–7. 20670305 10.1111/j.1651-2227.2010.01939.x

[14] Øien T, Schjelvaag A, Storrø O, Johnsen R, Simpson MR. Fish consumption at one year of age reduces the risk of eczema, asthma and wheeze at six years of age. Nutrients. 2019;11(9):1969. 31438628 10.3390/nu11091969 PMC6770937

[15] Klingberg S, Brekke HK, Ludvigsson J. Introduction of fish and other foods during infancy and risk of asthma in the All Babies In Southeast Sweden cohort study. Eur J Pediatrics. 2019;178(3):395–402. 10.1007/s00431-018-03312-5 PMC6437126 30617650

[16] Vale SL, Lobb M, Netting MJ, Murray K, Clifford R, Campbell DE, et al. A systematic review of infant feeding food allergy prevention guidelines - can we AGREE? World Allergy Organ J. 2021;14(6):100550. 10.1016/j.waojou.2021.100550 PMC8173304 34141050

[17] Skjerven HO, Lie A, Vettukattil R, Rehbinder EM, LeBlanc M, Asarnoj A, et al. Early food intervention and skin emollients to prevent food allergy in young children (PreventADALL): a factorial, multicentre, cluster-randomised trial. Lancet. 2022;399(10344):2398–411. 35753340 10.1016/S0140-6736(22)00687-0

[18] Barman M, Murray F, Bernardi AI, Broberg K, Bölte S, Hesselmar B, et al. Nutritional impact on Immunological maturation during Childhood in relation to the Environment (NICE): a prospective birth cohort in northern Sweden. BMJ Open. 2018;8(10):e022013. 30344169 10.1136/bmjopen-2018-022013 PMC6196815

[19] Stråvik M, Barman M, Hesselmar B, Sandin A, Wold AE, Sandberg AS. Maternal intake of cow’s milk during lactation is associated with lower prevalence of food allergy in offspring. Nutrients. 2020;12(12):3680. 10.3390/nu12123680 PMC7761074 33260602

[20] Williams HC, Burney PG, Hay RJ, Archer CB, Shipley MJ, Hunter JJ, et al. The U.K. working party’s diagnostic criteria for atopic dermatitis. I. Derivation of a minimum set of discriminators for atopic dermatitis. Br J Dermatol. 1994;131(3):383–96. 7918015 10.1111/j.1365-2133.1994.tb08530.x

[21] Williams HC, Burney PG, Strachan D, Hay RJ. The U.K. working party’s diagnostic criteria for atopic dermatitis. II. Observer variation of clinical diagnosis and signs of atopic dermatitis. Br J Dermatol. 1994;131(3):397–405. 7918016 10.1111/j.1365-2133.1994.tb08531.x

[22] Williams HC, Burney PG, Pembroke AC, Hay RJ. The U.K. Working Party’s Diagnostic Criteria for Atopic Dermatitis. III. Independent hospital validation. Br J Dermatol. 1994;131(3):406–16. 7918017 10.1111/j.1365-2133.1994.tb08532.x

[23] Peroni DG, Hufnagl K, Comberiati P, Roth-Walter F. Lack of iron, zinc, and vitamins as a contributor to the etiology of atopic diseases. Front Nutr. 2023;9:1032481. 10.3389/fnut.2022.1032481 PMC9869175 36698466

[24] Bono MR, Tejon G, Flores-Santibañez F, Fernandez D, Rosemblatt M, Sauma D. Retinoic acid as a modulator of T cell immunity. Nutrients. 2016;8(6):349. 10.3390/nu8060349 PMC4924190 27304965

[25] Barman M, Johansson S, Hesselmar B, Wold AE, Sandberg A-S, Sandin A. High levels of both n-3 and n-6 long-chain polyunsaturated fatty acids in cord serum phospholipids predict allergy development. PLoS ONE. 2013;8(7):e67920. 23874467 10.1371/journal.pone.0067920 PMC3707846

[26] Radzikowska U, Rinaldi AO, Çelebi Sözener Z, Karaguzel D, Wojcik M, Cypryk K, et al. The influence of dietary fatty acids on immune responses. Nutrients. 2019;11(12):2990. 10.3390/nu11122990 PMC6950146 31817726

[27] The Swedish Food. Agency food database, version 2025-10-29. Available from: https://soknaringsinnehall.livsmedelsverket.se/.

[28] Jonsson K, Barman M, Moberg S, Sjöberg A, Brekke HK, Hesselmar B, et al. Fat intake and breast milk fatty acid composition in farming and nonfarming women and allergy development in the offspring. Pediatr Res. 2016;79(1–1):114–23. 26389822 10.1038/pr.2015.187

[29] Sausenthaler S, Kompauer I, Borte M, Herbarth O, Schaaf B, Berg A, et al. Margarine and butter consumption, eczema and allergic sensitization in children. The LISA birth cohort study. Pediatr Allergy Immunol. 2006;17(2):85–93. 16618357 10.1111/j.1399-3038.2005.00366.x

[30] Roduit C, Frei R, Ferstl R, Loeliger S, Westermann P, Rhyner C, et al. High levels of butyrate and propionate in early life are associated with protection against atopy. Allergy. 2019;74(4):799–809. 30390309 10.1111/all.13660

[31] Swedish Food Agency. Good food for infants under one year. Uppsala: Swedish Food Agency; 2024.

[32] Wells HG, Osborne TB. The biological reactions of the vegetable proteins. J Infect Dis. 1911;8(1):66–124.

[33] Lamont AG, Mowat AM, Parrott DM. Priming of systemic and local delayed-type hypersensitivity responses by feeding low doses of ovalbumin to mice. Immunology. 1989;66(4):595–9. 2714839 PMC1385163

