# Supplementary material for: Food introduction during the first year of life in a Swedish birth cohort – associations with allergy at 6 years of age
Source: Nutr J. 2026 Jun 17;25:66. doi: 10.1186/s12937-026-01344-4 (PMC13274041; doi:10.1186/s12937-026-01344-4)
Supplement: Supplementary file 1 — Supplementary Material 1. [file 12937_2026_1344_MOESM1_ESM.docx]

Supplementary Materials

**Food introduction during the first year of life in a Swedish birth cohort – associations with allergy at 6 years of age**

Mia Stråvik ^1,*^, Anna Sandin ^2^, Bill Hesselmar ^3^, Agnes E. Wold ^4^, Malin Barman ^1^ and Ann-Sofie Sandberg ^1^

^1^ Department of Life Sciences, Food and Nutrition Science, Chalmers University of Technology, Gothenburg, Sweden

^2^ Department of Clinical Science, Pediatrics, Sunderby Research Unit, Umeå University, Umeå, Sweden

^3^ Department of Paediatrics, Institute of Clinical Sciences, Sahlgrenska Academy, University of Gothenburg, Gothenburg, Sweden

^4^ Department of Infectious Diseases, Institute of Biomedicine, Sahlgrenska Academy, University of Gothenburg, Gothenburg, Sweden

^*^Correspondence:
Mia Stråvik
mia.stravik@chalmers.se

**Content**

**Supplementary Tables**

**Supplementary Table 1.** Month of first food introduction by allergy diagnosis at six years of age.

**Supplementary Table 2.** Month of first food introduction among children without early food allergic symptoms, defined as absence of a food allergy diagnosis at one year, grouped by allergy diagnosis.

**Supplementary Table 3.** Logistic regression models of food allergy in relation to timing of food introduction.

**Supplementary Table 4.** Logistic regression models of atopic eczema in relation to timing of food introduction.

**Supplementary Table 5.** Logistic regression models of allergic asthma in relation to timing of food introduction.

**Supplementary Figures**

**Supplementary Fig. 1.** Monthly proportion of infants receiving formula during the first year of life.

**Supplementary Tables**

**Supplementary Table 1.** Month of first food introduction by allergy diagnosis at six years of age.

|  | **Month of age at food introduction**  Median (25^th^-75^th^ percentiles) | | | | | | | |
| --- | --- | --- | --- | --- | --- | --- | --- | --- |
|  | **All children *n =* 462** | **Non-allergic**  ***n =* 300** | **Food allergy**  ***n =* 16** | ***p*** | **Atopic eczema**  ***n =* 69** | ***p*** | **Allergic asthma**  ***n =* 52** | ***p*** |
| Fruits and/or fruit juice | 4.0 (4.0-5.0) | 4.0 (4.0-5.0) | 4.0 (3.25-5.0) | 0.159 | 4.0 (4.0-5.0) | 0.155 | 4.0 (4.0-5.0) | 0.469 |
| Potatoes and roots | 4.0 (4.0-5.0) | 4.0 (4.0-5.0) | 4.0 (4.0-5.0) | 0.491 | 4.0 (4.0-5.0) | 0.332 | 4.0 (4.0-5.0) | 0.491 |
| Berries | 5.0 (4.0-6.0) | 5.0 (4.0-7.0) | 5.0 (4.0-6.0) | 0.221 | 5.0 (4.0-7.0) | 0.781 | 5.0 (4.0-7.0) | 0.825 |
| Vegetable oils | 6.0 (5.0-7.0) | 5.0 (4.25-7.0) | 5.0 (4.25-6.0) | 0.445 | 5.0 (4.0-6.0) | 0.268 | 6.0 (4.0-7.0) | 0.862 |
| Butter and/or blended butter | 6.0 (4.0-7.0) | 6.0 (4.0-7.0) | 5.5 (4.0-9.75) | 0.916 | 6.0 (4.0-9.0) | 0.192 | 6.0 (5.0-8.0) | 0.128 |
| Fish | 6.0 (5.0-6.0) | 6.0 (5.0-6.0) | 6.0 (5.0-7.0) | 0.162 | 6.0 (5.0-7.0) | 0.621 | 6.0 (5.0-6.75) | 0.971 |
| Meat | 6.0 (5.0-6.0) | 6.0 (5.0-6.0) | 6.0 (5.25-8.0) | 0.041 | 6.0 (5.0-6.0) | 0.303 | 6.0 (5.0-6.0) | 0.234 |
| Bread and/or biscuits | 6.0 (5.0-7.0) | 6.0 (5.0-7.0) | 6.0 (5.0-7.0) | 0.960 | 6.0 (5.0-7.0) | 0.173 | 6.0 (5.0-8.0) | 0.602 |
| Egg | 7.0 (6.0-8.0) | 7.0 (5.0-8.0) | 7.0 (6.25-11.25) | 0.049 | 7.0 (5.0-8.5) | 0.681 | 7.0 (6.0-8.0) | 0.334 |
| Yoghurt | 8.0 (6.0-11.0) | 7.0 (6.0-10.0) | 8.5 (5.25-11.75) | 0.520 | 8.0 (5.5-12.0) | 0.121 | 8.0 (5.0-12.0) | 0.668 |
| Cow’s milk | 8.0 (6.0-11.0) | 8.0 (6.0-11.0) | 9.0 (7.25-12.0) | 0.098 | 9.0 (6.0-12.0) | 0.079 | 8.0 (6.0-11.0) | 0.958 |
| Sour milk (fil) | 11.0 (8.0-12.0) | 11.0 (7.0-12.0) | 12.0 (10.0-12.0) | 0.210 | 12.0 (8.0-12.0) | 0.066 | 12.0 (8.0-12.0) | 0.993 |
| Margarine | 12.0 (9.0-12.0) | 12.0 (9.0-12.0) | 9.0 (5.0-12.0) | 0.021 | 12.0 (8.0-12.0) | 0.170 | 12.0 (9.0-12.0) | 0.759 |
| Ice cream | 12.0 (9.0-12.0) | 12.0 (9.0-12.0) | 12.0 (9.25-12.0) | 0.471 | 12.0 (10.5-12.0) | 0.048 | 12.0 (9.5-12.0) | 0.110 |
| Nuts and/or almonds | 12.0 (10.0-12.0) | 12.0 (10.0-12.0) | 12.0 (12.0-12.0) | 0.229 | 12.0 (10.0-12.0) | 0.665 | 12.0 (9.0-12.0) | 0.388 |
| Peanuts and/or peanut oil | 12.0 (11.0-12.0) | 12.0 (11.0-12.0) | 12.0 (12.0-12.0) | 0.061 | 12.0 (10.0-12.0) | 0.509 | 12.0 (12.0-12.0) | 0.626 |
| Probiotic-containing foods/drinks | 12.0 (11.0-12.0) | 12.0 (11.25-12.0) | 12.0 (10.5-12.0) | 0.869 | 12.0 (12.0-12.0) | 0.780 | 12.0 (12.0-12.0) | 0.914 |

The numbers represent age when consumption occurred, not age when questionnaire was completed (i.e., 0 = introduction in past month before turning one month). Differences between children with an allergy diagnosis and children without allergies or allergic sensitization (i.e., non-allergic group) were tested using the Mann-Whitney U test.

**Supplementary Table 2.** Month of first food introduction among children without early food allergic symptoms, defined as absence of a food allergy diagnosis at one year, grouped by allergy diagnosis.

|  | **Month of age at food introduction**  Median (25^th^-75^th^ percentiles) | | | | | | | |
| --- | --- | --- | --- | --- | --- | --- | --- | --- |
|  | **All children *n =* 386** | **Non-allergic**  ***n =* 266** | **Food allergy**  ***n =* 6** | ***p*** | **Atopic eczema**  ***n =* 43** | ***p*** | **Allergic asthma**  ***n =* 39** | ***p*** |
| Fruits and/or fruit juice | 4.0 (4.0-5.0) | 4.0 (4.0-5.0) | 4.0 (3.0-5.25) | 0.390 | 4.0 (4.0-5.0) | 0.260 | 4.0 (4.0-5.0) | 0.795 |
| Potatoes and roots | 4.0 (4.0-5.0) | 4.0 (4.0-5.0) | 4.0 (3.75-5.0) | 0.830 | 4.0 (4.0-5.0) | 0.527 | 4.0 (4.0-5.0) | 0.906 |
| Berries | 5.0 (4.0-6.0) | 5.0 (4.0-6.25) | 5.0 (4.5-6.5) | 0.881 | 5.0 (4.0-7.0) | 0.679 | 5.0 (4.0-6.0) | 0.911 |
| Vegetable oils | 5.5 (5.0-7.0) | 5.0 (4.0-7.0) | 5.5 (4.75-9.5) | 0.564 | 5.0 (4.0-6.0) | 0.632 | 6.0 (5.0-9.0) | 0.064 |
| Butter and/or blended butter | 6.0 (4.0-7.0) | 5.0 (4.0-7.0) | 4.0 (3.0-7.0) | 0.122 | 6.0 (4.0-7.0) | 0.962 | 6.0 (4.0-8.0) | 0.483 |
| Fish | 6.0 (5.0-6.0) | 6.0 (5.0-6.0) | 7.0 (5.75-9.25) | 0.030 | 5.0 (4.0-6.0) | 0.208 | 6.0 (5.0-6.0) | 0.933 |
| Meat | 6.0 (5.0-6.0) | 6.0 (5.0-6.0) | 7.5 (6.0-9.75) | 0.005 | 5.0 (4.0-6.0) | 0.106 | 6.0 (5.0-6.0) | 0.492 |
| Bread and/or biscuits | 6.0 (5.0-7.0) | 6.0 (5.0-7.0) | 5.5 (4.0-7.0) | 0.675 | 6.0 (5.0-8.0) | 0.430 | 6.0 (5.0-8.0) | 0.795 |
| Egg | 7.0 (5.0-8.0) | 7.0 (5.0-8.0) | 7.0 (6.75-9.75) | 0.276 | 6.0 (5.0-8.0) | 0.600 | 7.0 (6.0-8.0) | 0.554 |
| Yoghurt | 7.0 (6.0-10.0) | 7.0 (6.0-10.0) | 6.0 (4.75-8.25) | 0.207 | 7.0 (5.0-10.0) | 0.667 | 7.0 (5.0-10.0) | 0.435 |
| Cow’s milk | 8.0 (6.0-11.0) | 8.0 (6.0-10.0) | 8.5 (7.75-9.75) | 0.284 | 8.0 (6.0-10.0) | 0.684 | 7.0 (5.0-9.0) | 0.290 |
| Sour milk (fil) | 10.0 (7.0-12.0) | 11.0 (7.0-12.0) | 10.0 (6.25-12.0) | 0.760 | 12.0 (8.0-12.0) | 0.373 | 9.0 (7.0-12.0) | 0.715 |
| Margarine | 12.0 (9.0-12.0) | 12.0 (9.0-12.0) | 9.0 (6.25-12.0) | 0.098 | 12.0 (8.0-12.0) | 0.445 | 12.0 (10.0-12.0) | 0.943 |
| Ice cream | 12.0 (9.0-12.0) | 12.0 (9.0-12.0) | 11.0 (6.25-12.0) | 0.636 | 12.0 (9.0-12.0) | 0.318 | 12.0 (9.0-12.0) | 0.116 |
| Nuts and/or almonds | 12.0 (10.0-12.0) | 12.0 (10.0-12.0) | 12.0 (11.0-12.0) | 0.503 | 12.0 (9.0-12.0) | 0.644 | 12.0 (9.0-12.0) | 0.574 |
| Peanuts and/or peanut oil | 12.0 (11.0-12.0) | 12.0 (11.0-12.0) | 12.0 (11.5-12.0) | 0.492 | 12.0 (10.0-12.0) | 0.353 | 12.0 (12.0-12.0) | 0.693 |
| Probiotic-containing foods/drinks | 12.0 (11.0-12.0) | 12.0 (11.0-12.0) | 11.0 (3.0-12.0) | 0.147 | 12.0 (10.0-12.0) | 0.484 | 12.0 (12.0-12.0) | 0.592 |

The numbers represent age when consumption occurred, not age when questionnaire was completed (i.e., 0 = introduction in past month before turning one month). Differences between children without early food allergic symptoms within each allergy group and children without any allergic symptoms (i.e., non-allergic group) were tested using the Mann-Whitney U test. Children with a food allergy diagnosis at one year (*n =* 35), uncertain food allergies or avoidance due to suspected food allergies at one year (*n =* 13), and children without information on allergy status at one year (*n =* 28) were excluded to take reverse causation into account.

**Supplementary Table 3.** Logistic regression models of food allergy in relation to timing of food introduction.

|  | **Crude** | | |  | **Adjusted ^1^** | | |
| --- | --- | --- | --- | --- | --- | --- | --- |
|  | **OR (95% CI)** | ***p*** | **FDR** |  | **OR (95% CI)** | ***p*** | **FDR** |
| Berries | 1.277 (0.939-1.869) | 0.163 | 0.423 |  | 1.235 (0.891-1.831) | 0.250 | 0.452 |
| Bread and/or biscuits | 1.067 (0.833-1.425) | 0.633 | 0.633 |  | 1.091 (0.836-1.487) | 0.551 | 0.678 |
| Butter and/or blended butter | 0.924 (0.755-1.159) | 0.463 | 0.596 |  | 0.959 (0.771-1.229) | 0.722 | 0.764 |
| Cow's milk | 0.862 (0.705-1.039) | 0.128 | 0.423 |  | 0.900 (0.733-1.092) | 0.296 | 0.484 |
| Eggs | 0.799 (0.648-0.987) | 0.035 | 0.215 |  | 0.844 (0.680-1.048) | 0.120 | 0.452 |
| Fish | 0.864 (0.675-1.158) | 0.279 | 0.457 |  | 0.820 (0.630-1.108) | 0.160 | 0.452 |
| Fruits and/or fruit juice | 1.412 (0.905-2.383) | 0.166 | 0.423 |  | 1.417 (0.880-2.478) | 0.190 | 0.452 |
| Ice cream | 0.943 (0.734-1.144) | 0.596 | 0.631 |  | 0.970 (0.750-1.190) | 0.792 | 0.792 |
| Margarine | 1.263 (1.052-1.512) | 0.010 | 0.187 |  | 1.287 (1.069-1.549) | 0.007 | 0.122 |
| Meat | 0.770 (0.610-1.003) | 0.036 | 0.215 |  | 0.758 (0.597-0.987) | 0.028 | 0.249 |
| Nuts and/or almonds | 0.739 (0.398-1.045) | 0.188 | 0.423 |  | 0.776 (0.425-1.085) | 0.251 | 0.452 |
| Peanuts and/or peanut oil | 0.554 (0.138-0.939) | 0.159 | 0.423 |  | 0.569 (0.145-0.960) | 0.169 | 0.452 |
| Potatoes and roots | 1.309 (0.719-2.507) | 0.397 | 0.556 |  | 1.338 (0.716-2.629) | 0.379 | 0.524 |
| Probiotic-containing foods/drinks | 1.041 (0.891-1.176) | 0.558 | 0.628 |  | 1.041 (0.891-1.179) | 0.565 | 0.678 |
| Sour milk (fil) | 0.896 (0.714-1.075) | 0.279 | 0.457 |  | 0.907 (0.717-1.094) | 0.353 | 0.524 |
| Vegetable oils | 1.174 (0.925-1.536) | 0.219 | 0.439 |  | 1.177 (0.920-1.566) | 0.232 | 0.452 |
| Yoghurt | 0.941 (0.780-1.132) | 0.516 | 0.619 |  | 0.954 (0.786-1.159) | 0.628 | 0.706 |

^1^ Adjusted for allergic heredity (parent and/or siblings).
Logistic regression models between month of food introduction (continuous, reversed order) and food allergy at six years (no/yes). Odds ratios (ORs) with 95% profile likelihood confidence intervals (CIs) are shown. *P*-values were calculated using the Wald test and adjusted for the false discovery rate (FDR) using the Benjamini-Hochberg procedure. An OR < 1 indicates lower odds of food allergy following an earlier introduction. Crude: *n =* 16 with food allergy, *n =* 300 without any allergies; Adjusted: *n =* 15 with food allergy, *n =* 283 without any allergies.

**Supplementary Table 4.** Logistic regression models of atopic eczema in relation to timing of food introduction.

|  | **Crude** | | |  | **Adjusted ^1^** | | |
| --- | --- | --- | --- | --- | --- | --- | --- |
|  | **OR (95% CI)** | ***p*** | **FDR** |  | **OR (95% CI)** | ***p*** | **FDR** |
| Berries | 0.974 (0.857-1.118) | 0.702 | 0.847 |  | 0.938 (0.816-1.085) | 0.376 | 0.667 |
| Bread and/or biscuits | 0.955 (0.841-1.090) | 0.485 | 0.794 |  | 0.944 (0.826-1.082) | 0.398 | 0.667 |
| Butter and/or blended butter | 0.896 (0.804-1.001) | 0.049 | 0.382 |  | 0.897 (0.799-1.009) | 0.066 | 0.593 |
| Cow's milk | 0.919 (0.834-1.011) | 0.085 | 0.382 |  | 0.928 (0.839-1.025) | 0.145 | 0.627 |
| Eggs | 0.958 (0.857-1.073) | 0.452 | 0.794 |  | 0.965 (0.861-1.084) | 0.541 | 0.812 |
| Fish | 1.004 (0.861-1.185) | 0.963 | 0.963 |  | 0.977 (0.830-1.163) | 0.786 | 0.962 |
| Fruits and/or fruit juice | 1.119 (0.910-1.409) | 0.312 | 0.625 |  | 1.102 (0.886-1.404) | 0.408 | 0.667 |
| Ice cream | 0.922 (0.816-1.027) | 0.163 | 0.488 |  | 0.935 (0.825-1.047) | 0.268 | 0.667 |
| Margarine | 1.097 (0.987-1.214) | 0.079 | 0.382 |  | 1.105 (0.992-1.228) | 0.066 | 0.593 |
| Meat | 1.051 (0.889-1.267) | 0.579 | 0.847 |  | 1.034 (0.871-1.250) | 0.716 | 0.962 |
| Nuts and/or almonds | 1.010 (0.885-1.139) | 0.877 | 0.963 |  | 1.001 (0.869-1.138) | 0.990 | 0.990 |
| Peanuts and/or peanut oil | 1.063 (0.951-1.180) | 0.262 | 0.589 |  | 1.062 (0.944-1.186) | 0.300 | 0.667 |
| Potatoes and roots | 1.065 (0.796-1.448) | 0.680 | 0.847 |  | 1.019 (0.750-1.404) | 0.908 | 0.962 |
| Probiotic-containing foods/drinks | 0.996 (0.915-1.075) | 0.931 | 0.963 |  | 0.993 (0.911-1.073) | 0.873 | 0.962 |
| Sour milk (fil) | 0.921 (0.831-1.012) | 0.098 | 0.382 |  | 0.928 (0.835-1.023) | 0.147 | 0.627 |
| Vegetable oils | 1.076 (0.957-1.221) | 0.234 | 0.589 |  | 1.073 (0.951-1.222) | 0.268 | 0.667 |
| Yoghurt | 0.925 (0.841-1.016) | 0.106 | 0.382 |  | 0.934 (0.846-1.030) | 0.174 | 0.627 |

^1^ Adjusted for allergic heredity (parent and/or siblings).

Logistic regression models between month of food introduction (continuous, reversed order) and atopic eczema at six years (no/yes). Odds ratios (ORs) with 95% profile likelihood confidence intervals (CIs) are shown. *P*-values were calculated using the Wald test and adjusted for the false discovery rate (FDR) using the Benjamini-Hochberg procedure. An OR < 1 indicates lower odds of atopic eczema following an earlier introduction. Crude: *n =* 69 with atopic eczema, *n =* 300 without any allergies; Adjusted: *n =* 66 with atopic eczema, *n =* 283 without any allergies.

**Supplementary Table 5.** Logistic regression models of allergic asthma in relation to timing of food introduction.

|  | **Crude** | | |  | **Adjusted ^1^** | | |
| --- | --- | --- | --- | --- | --- | --- | --- |
|  | **OR (95% CI)** | ***p*** | **FDR** |  | **OR (95% CI)** | ***p*** | **FDR** |
| Berries | 0.973 (0.843-1.136) | 0.718 | 0.901 |  | 0.951 (0.816-1.121) | 0.534 | 0.939 |
| Bread and/or biscuits | 0.968 (0.841-1.125) | 0.663 | 0.901 |  | 0.982 (0.846-1.152) | 0.821 | 0.939 |
| Butter and/or blended butter | 0.891 (0.788-1.011) | 0.067 | 0.901 |  | 0.892 (0.783-1.019) | 0.087 | 0.939 |
| Cow's milk | 0.995 (0.893-1.110) | 0.934 | 0.934 |  | 1.014 (0.907-1.134) | 0.806 | 0.939 |
| Eggs | 0.955 (0.838-1.091) | 0.487 | 0.901 |  | 0.951 (0.834-1.088) | 0.459 | 0.939 |
| Fish | 1.023 (0.856-1.248) | 0.810 | 0.901 |  | 0.996 (0.827-1.221) | 0.969 | 0.969 |
| Fruits and/or fruit juice | 1.038 (0.836-1.324) | 0.751 | 0.901 |  | 1.022 (0.816-1.315) | 0.856 | 0.939 |
| Ice cream | 0.925 (0.804-1.044) | 0.237 | 0.901 |  | 0.933 (0.809-1.059) | 0.311 | 0.939 |
| Margarine | 0.986 (0.857-1.119) | 0.831 | 0.901 |  | 0.990 (0.860-1.127) | 0.887 | 0.939 |
| Meat | 1.187 (0.956-1.518) | 0.147 | 0.901 |  | 1.172 (0.943-1.504) | 0.182 | 0.939 |
| Nuts and/or almonds | 1.070 (0.934-1.211) | 0.306 | 0.901 |  | 1.080 (0.940-1.227) | 0.255 | 0.939 |
| Peanuts and/or peanut oil | 0.979 (0.842-1.114) | 0.761 | 0.901 |  | 0.985 (0.844-1.127) | 0.831 | 0.939 |
| Potatoes and roots | 1.181 (0.834-1.699) | 0.358 | 0.901 |  | 1.184 (0.825-1.724) | 0.369 | 0.939 |
| Probiotic-containing foods/drinks | 1.019 (0.932-1.103) | 0.652 | 0.901 |  | 1.013 (0.925-1.098) | 0.771 | 0.939 |
| Sour milk (fil) | 1.010 (0.911-1.113) | 0.851 | 0.901 |  | 1.024 (0.923-1.130) | 0.652 | 0.939 |
| Vegetable oils | 0.976 (0.866-1.107) | 0.697 | 0.901 |  | 0.980 (0.869-1.113) | 0.752 | 0.939 |
| Yoghurt | 0.967 (0.869-1.077) | 0.541 | 0.901 |  | 0.976 (0.874-1.090) | 0.662 | 0.939 |

^1^ Adjusted for allergic heredity (parent and/or siblings).
Logistic regression models between month of food introduction (continuous, reversed order) and allergic asthma at six years (no/yes). Odds ratios (ORs) with 95% profile likelihood confidence intervals (CIs) are shown. *P*-values were calculated using the Wald test and adjusted for the false discovery rate (FDR) using the Benjamini-Hochberg procedure. An OR < 1 indicates lower odds of allergic asthma following an earlier introduction. Crude: *n =* 52 with allergic asthma, *n =* 300 without any allergies; Adjusted: *n =* 51 with allergic asthma, *n =* 283 without any allergies.

**Supplementary Figures**

**
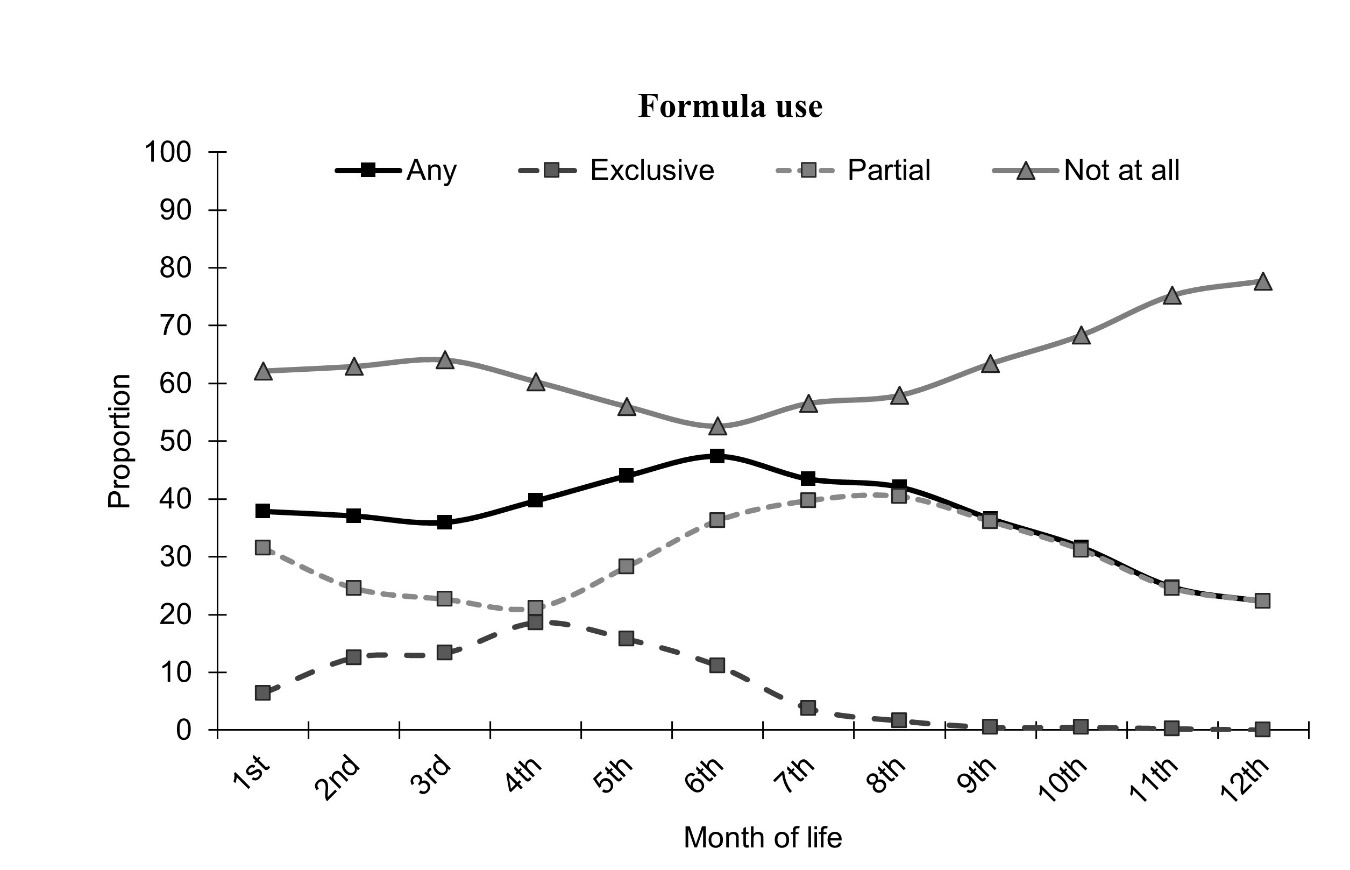
**

**Supplementary Fig. 1.** Monthly proportion of infants receiving formula during the first year of life.
